# A scoping review of emotion regulation and inhibition in emotional eating and binge-eating disorder: what about a continuum?

**DOI:** 10.1186/s40337-023-00916-7

**Published:** 2023-11-10

**Authors:** Mahé Arexis, Gilles Feron, Marie-Claude Brindisi, Pierre-Édouard Billot, Stéphanie Chambaron

**Affiliations:** 1 Centre Des Sciences du Goût Et de L’Alimentation, CNRS, INRAE, Institut Agro, Université de Bourgogne, 21000 Dijon, France; 2 Université de Franche-Comté, LINC, 25000 Besançon, France; 3 Centre Spécialisé de L’Obésité Bourgogne, Centre Hospitalier Universitaire Dijon Bourgogne, Dijon, France

**Keywords:** Binge-eating disorder, Emotional eating, Overeating, Emotion regulation, Inhibition, Scoping review

## Abstract

**Background:**

Emotional eating is defined as a nonpathological eating behavior, whereas binge-eating disorder (BED) is defined as a pathological eating behavior. While different, both share some striking similarities, such as deficits in emotion regulation and inhibition. Previous research has suggested the existence of an “eating continuum” that might reflect the increased severity of overeating behaviors, that is, from nonpathological overeating to BED. The main aims of this scoping review were to explore in the literature the idea of a continuum between emotional eating and BED and to observe whether deficits in emotion regulation and inhibition follow this continuum in terms of severity. The other aims were to hopefully clarify the ill-defined concept of overeating, to question the potential role of positive emotions and to identify potential knowledge gaps.

**Method:**

A systematic scoping review was conducted following the Preferred Reporting Items for Systematic reviews and Meta-Analyses extension for Scoping Reviews (PRISMA-ScR) guidelines. Two databases (PubMed/Medline and PsycINFO) were examined in complete accordance with the beforehand sharply defined eligibility and exclusion criteria. The main criteria included adults (≥ 18) with emotional eating, BED or overeating and emotion regulation and inhibition as exposure criteria.

**Results:**

Thirty-two studies were included in this scoping review. If the results showed a link between emotional eating and BED, with the presence of inhibition and emotion regulation deficits in both eating behaviors, no mention of a continuum between emotional eating and BED was found.

**Conclusion:**

In the absence of research directly comparing emotional eating and BED in the same studies and testing the potential increase in severity of emotion regulation and inhibition deficits along this continuum, there is currently no certainty that a continuum exists between emotional eating and BED. In the end, the idea of a continuum in terms of increased severity of overeating and in terms of emotion regulation and inhibition deficits between emotional eating and BED appears to be a gap in knowledge in the literature. This scoping review highlights the need for further research to identify knowledge gaps.

## Abstract (plain language summary)

Emotional eating (EE) is defined as a nonpathological eating behavior, whereas binge-eating disorder (BED) is defined as a pathological eating behavior. While different, both share some striking similarities, such as deficits in emotion regulation (ER) and inhibition. Previous research has suggested the existence of an “eating continuum” that might reflect the increased severity of overeating behaviors, that is, from nonpathological overeating to BED. The main aims of this scoping review were to explore in the literature the idea of a continuum between EE and BED and to observe whether deficits in ER and inhibition follow this continuum in terms of severity. A systematic scoping review was conducted, and thirty-two studies were included in this review. If the results showed a link between EE and BED, with the presence of inhibition and ER deficits in both eating behaviors, no mention of a continuum between EE and BED, or in relation to a continuum, was found. Thus, in the absence of research directly comparing EE and BED in the same studies and testing the potential increase in severity of ER and inhibition deficits along this continuum, there is currently no certainty about the existence or absence of such a continuum.

## Introduction

Our scoping review mainly focused on emotional eating (EE) and binge-eating disorder (BED). EE is an eating behavior that can be defined as “the tendency to overeat in response to negative emotions […]” ([1], p. 106) but in a nonpathological way. It differs from BED, which was formally indexed in 2013 in the DSM-5 as a discrete eating disorder. BED symptoms include recurrent binge-eating episodes (i.e., eating a larger amount of food than most people do during a discrete period of time, with at least one episode per week for three months), “a sense of lack of control over eating during the episode” and “marked distress regarding binge eating”, but without compensatory behaviors as in anorexia nervosa or bulimia nervosa [2].

Although different, both EE and BED appear to be affected by deficits in emotion regulation (ER) and inhibition [3–10]. Indeed, both individuals with EE and BED present with overeating behaviors caused by emotion regulation difficulties and a lack of inhibition/greater impulsivity. For example, it has been shown that negative emotions act as a trigger for binge-eating episodes in BED [7, 11, 12], and some data also suggest that positive emotions may increase food consumption [7]. Binge eating can be seen as a way to regulate negative emotions (but it is yet uncertain if this strategy successfully improves mood, see Leehr et al. and Stein et al. [7, 13]), and BED patients are more prone to use maladaptive strategies such as suppression or rumination [4].

**Fig. 1:**
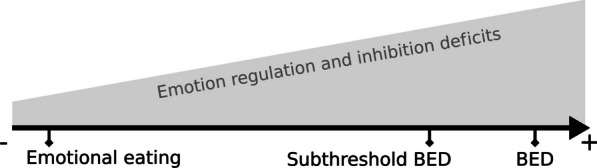
Schematic view of increased emotion regulation and inhibition deficits along a continuum between nonpathological emotional eating and binge-eating disorder (BED)

Davis [14] suggested the existence of an “(over) eating continuum”: in some way, on one end of the continuum are nonpathological overeating behaviors and at the other end is BED, which is a pathological and extreme state of overeating. The evolution on this continuum, therefore, reflects the “increased severity and compulsiveness” of overeating behaviors. It is also important to emphasize that this idea of a continuum in severity and compulsiveness between those eating behaviors is also reported by clinicians and physicians. It is therefore reasonable to think that the severity of ER and inhibition deficits could increase along this continuum between EE and BED, as shown in Fig. 1. Taken together, this information is a starting point to lead a systematic screening of the literature. Since our main goals are to clarify the concept of continuum and to identify knowledge gaps, we chose to conduct a scoping review following the guidelines of PRISMA-ScR (Preferred Reporting Items for Systematic reviews and Meta-Analyses extension for Scoping Reviews) [15].

The present scoping review aimed (1) to address the possible existence of a continuum between EE and BED; (2) to address the possibility of an increase in the severity of deficits in emotion regulation and inhibition; and (3) to address the ill-defined concept of overeating. Indeed, is overeating (OE) a symptom, an eating behavior, a synonymous concept of EE, or a synonym for binge eating? (4) The final aim was to potentially investigate whether positive emotions can, like negative emotions, trigger emotional eating episodes associated with emotion regulation and/or inhibition difficulties. Finally, this scoping review also aimed to identify gaps in knowledge.

## Method

The scoping review was conducted following the Preferred Reporting Items for Systematic reviews and Meta-Analyses extension for Scoping Reviews (PRISMA-ScR) guidelines [15].

The review protocol can be accessed at HAL (https://hal.science/hal-03643357v1—HAL Id/Registration number: hal-03643357) [16].

### Eligibility criteria

Studies were selected based on the following criteria:

The PICOS framework was used to highlight the main criteria. PICOS criteria: *Populations*: People (adult human subjects ≥ 18) with binge-eating disorder (BED) (and meeting the full DSM-IV-TR or DSM-5 criteria for BED) or subthreshold BED and people (adult human subjects, 18 +) presenting with emotional eating (EE) or emotional overeating (EO). *Interventions/Exposures*: Our review focused on the impact of “emotion regulation” and “inhibitory control” on BED and EE. *Comparisons*: Our review did not focus on studies with specific comparisons. *Outcomes*: Our review considered all types of outcomes related to emotion regulation and inhibitory control in BED, EE, and EO (*e.g.*, deficit, level of attention, response impairment, and degree of compulsivity). *Studies*: All types of journal articles published in peer-reviewed scientific journals, either written in English or in French. The exclusion criteria were all types of reviews, book chapters, abstracts, preprints, theses, and articles focusing on therapies/treatments.

Only papers published between January 2009 and January 2022 were eligible for consideration. Although the MeSH (Medical Subject Headings) terms for binge-eating disorder were not introduced until 2010, the year 2009 was chosen because it was a “transition year” between the previous indexing of binge-eating disorder as bulimia nervosa and the introduction of the MeSH term BED in 2010.

### Information sources and search

Two electronic bibliographic databases, PubMed/Medline and PsycINFO, were searched to identify references related to the scoping review topic. The search focused on articles published between January 2009 and January 2022. The following search equation was used in both databases: ("Binge-Eating Disorder"[Mesh] OR BED OR Binge eater OR Emotional Eating OR Emotional Overeating OR Overeater OR Emotional eater OR Overeating) AND ("Emotional Regulation"[Mesh] OR Emotion regulation OR Reappraisal OR Rumination OR Attentional deployment OR Mood regulation OR "Inhibition, Psychological"[Mesh] OR Inhibitory control).

This database search stage was conducted by one of the authors, M. A. No additional references were added from other sources at this stage.

### Selection of sources of evidence

**Fig. 2:**
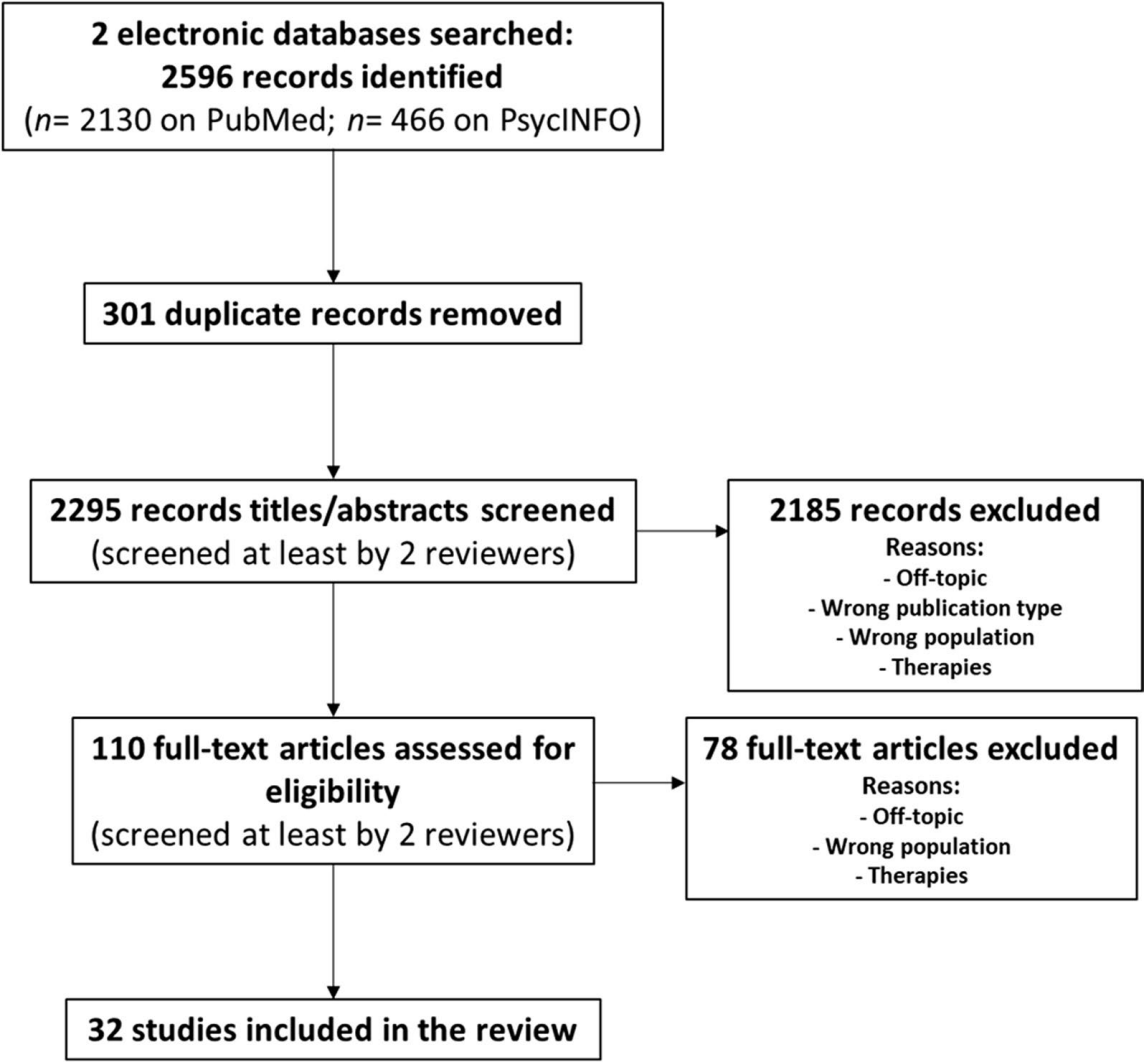
Flowchart of the literature search and screening/study selection process

Duplicates were removed, and all references were imported into Rayyan, an online application for systematic reviews [17]. Figure 2 shows the flowchart of the literature search and screening/study selection process. During the successive screening stages, at least 2 authors (M. A., and P.-E. B. or S. C., up to 4 authors, M. A., P.-E. B., S. C. and M.-C. B.) screened each record. Disagreements regarding study selection were resolved by a third or even a fourth investigator, and discussions took place between the authors. In the first screening step, for each article, the inclusion criteria described in Sect. "Eligibility criteria" were applied to both titles and abstracts. In the second screening step (eligibility), for each article, the inclusion criteria described in Sect. "Eligibility criteria" were applied to the entire article (i.e., a complete reading of the article). Note that at this stage, we screened the bibliographic references of the included articles to identify potential new references. At the end of this screening, no new articles were included.

### Data charting process and data items

Three investigators (authors M. A., P.-E. B., and S. C.) developed a data charting form to extract the relevant data from each of the 32 included studies. For each study, author M. A. extracted the data according to the following variables of the chart: authors, title, year of publication, country of the study, journal of publication, type of study, method/study design, participants and sample sizes, type of interventions, comparisons, and outcomes/results. Table 1 shows the final version of the chart with the main characteristics of the studies included in the scoping review. We did not include in Table 1 the journal of publication or type of study variables of the chart, as they were deemed irrelevant for this table. Note that we added in Table 1 a new column “Outcomes/Results highlight” with a simplified overview of the results, as well as a “Limitations” column.

**Table 1:** Characteristics of the studies included in the scoping review

Study	Participants	Method	Interventions/ Exposures	Highlights	Outcomes	Limitations
Braden et al.[22] (USA)	Adults living with overweight/obesity (n = 188) (Emotional Eaters and different types of EE among them)	Questionnaires / Scales	Emotion regulation	ER difficulties positively correlated with EE in response to depression, anxiety and boredom EE in response to depression associated with ER difficulties (regression analysis)	"[…] eating in response to positive emotions was not significantly related to poorer psychological well-being, greater eating disorder symptoms, or emotion dysregulation." "Exploratory analyses suggest possible unique relationships between types of emotional eating and specific facets of emotion regulation." "It is possible that when experiencing low intensity negative emotions (e.g., depression, boredom), an inability to attend to alternative, adaptive tasks may increase vulnerability for eating as a strategy for regulating negative affect." "Impulse control difficulties were closely related to the tendency to eat in response to depression and anxiety/anger which suggests that these two types of emotional eating may share an underlying impairment in inhibition." "[…] findings suggest that certain emotion regulation strategies may be more closely linked to various types of emotional eating."	- Only self-report measures (questionnaires/scales) - Specific EE population of adults with overweight or obesity: may not be representative of emotional eaters without overweight or obesity
Leehr et al. [25] (Germany)	Overweight participants with BED (BED +) (n = 24); Overweight controls without BED (BED-) (n = 23); Normal weight healthy controls (NWC) (n = 26)	Questionnaires / Scales Eye-tracking Anti-saccade task EEG	Inhibitory control Emotion regulation	Impulsivity and ER difficulties in BED > other groups EEG: Conflict processing less thorough in BED and NWC group	"Overall, results support the assumption of inhibitory control deficiencies in BED on a behavioral level." "Participants with BED reported higher impulsivity, trait craving, and lower emotion regulation capacities." "[…] the BED + sample committed significantly more errors than the NWC sample, suggesting inhibitory control difficulties in the BED + sample, which might be food-specific […]." "From a clinical perspective eating behavior of the three groups can be seen on a continuum from normal eating behavior to overeating, to binge eating." "Participants with BED reported higher impulsivity and lower emotion regulation capacities. The combined investigation of electrocortical processes and behavior contributes to an advanced understanding of behavioral and electrocortical processes underlying inhibitory control in BED."	- Mood was not directly measured before the mood induction procedure (before/after induction comparisons are not possible) - The mood induction procedure induced negative mood in general (no specific facets) - Only women among participants
Preuss et al. (2019) (Germany)	Overweight individuals with BED (n = 24) Overweight individuals without BED (n = 47) Healthy control group (n = 30)	Food Stroop task (FST) Door Opening task Stop Signal task Questionnaires / Scales	Inhibitory control	Deficits in interference inhibition in the FST: BED > healthy controls Performance on interference inhibition: BED < healthy controls	"Outpatients with BED showed more deficits in interference inhibition and by trend also in decision-making […]" "[…] poorer performance on interference inhibition […] in outpatients with BED compared to healthy controls but not compared to those without BED." "[…] absence of significant impairments in response inhibition […] in outpatients with overweight and disinhibited eating compared to healthy individuals."	- Very specific inclusion criteria: participants "had to report the therapeutic objective of weight reduction and at least two long-term unsuccessful weight maintenance efforts " - Unequal balance of sample size between groups - Unequal balance of gender among participants
Walenda et al. [27] (Poland)	Women with BED (n = 35) Healthy women (n = 41)	Questionnaires / Scales	Emotion regulation	Nonacceptance of emotional responses, lack of emotional clarity, difficulties engaging in goal-directed behavior, impulse control difficulties, and limited access to emotion regulation strategies: BED > healthy controls Rumination and self-blame (negative strategies): BED > healthy controls	"[…] a higher nonacceptance of emotional responses, lack of emotional clarity, difficulties engaging in goal-directed behavior, impulse control difficulties, and limited access to emotion regulation strategies were observed in the BED group compared to healthy women." "When it comes to negative strategies, in the present study women from clinical group were more inclinated to use rumination and self-blame (strong effects) than healthy women." "[…] important and still insufficiently understood role of emotional dysregulation in the clinical picture of this disorder"	- Only self-report measures (questionnaires, scales) - Self-report measures are mainly retrospective: possibility of recall biases - Only women among participants
Hege et al. (2015) (Germany)	Obese and overweight women with BED (n = 17—Overweight 4 / Obese 13) BMI-matched control group (n = 17—Overweight 3 / Obese 14)	Questionnaires / Scales Go / No-go task Magnetoencephalography (MEG)	Impulsivity Response inhibition	BIS-11 attentional impulsiveness scores: BED individuals > overweight and obese controls Decreased response inhibition performance in individuals with BED Individuals with BED: Hypoactivity in the prefrontal control network	"Specifically, increased attentional impulsiveness (a subscale of the BIS-11) in BED was related to decreased response inhibition performance and hypoactivity in the prefrontal control network, which was activated when response inhibition was required." "[…] attentional impulsiveness-related attenuation in response inhibition performance in individuals with BED." "[…] participants with BED showed a trend for a food-specific inhibition performance decline."	- Inclusion of participants with depressive symptoms and using antidepressant medication - Small sample sizes - Only women among participants
Loeber et al. [34] (Germany)	Obese women with BED (n = 17) Obese women without BED or any other eating disorder (n = 20) Normal-weight healthy control women (n = 20)	Questionnaires/Scales Go / No-go paradigm (computer-based experimental task)	Response inhibition Impulsivity	Obese BED: Less impairment of response inhibition to food-associated than to control stimuli Normal-Weight healthy control: pattern reversed No difference for obese participants	"[…] obese BED participants were better at inhibiting their responses to food associated than to neutral stimuli." "[…] restrained eating and mood are factors that moderate response inhibition to food-associated stimuli in obese patients with BED. […] apart from negative mood, positive mood might as well be a trigger for loss of control over eating behaviour."	- Go / No-go task only performed with food-related stimuli and no other rewarding stimuli - Mood was not systematically manipulated - Small sample sizes - Only women among participants
Grant & Chamberlain [28] (USA)	Young adults with BED (n = 17) Healthy controls (n = 17)	Questionnaires / Scales Cambridge Neuropsychological Test Automated Battery (CANTAB) A computerised version of the Wisconsin Card Sorting task Stop-Signal task One-Touch Stockings of Cambridge task (OTS) Cambridge Gamble Task (CGT) Spatial Working Memory Task (SWM)	Motor disinhibition Impulsivity	Impairments in stop-signal response inhibition (Stop-Signal Task) and executive planning (Stockings of Cambridge Task) in BED group > to healthy controls	"Binge-eating disorder was associated with impaired response inhibition and executive planning."	- Comorbidities were not excluded nor controlled in the BED group - Unequal balance of gender among participants - Small sample sizes
Munsch et al. [35] (Switzerland)	Female obese patients with BED (n = 22)	Questionnaires / Scales EMA (Ecological Momentary Assessment)	Emotion regulation	During binge days compared with non-binge days: Values for negative mood: ↗ Values for positive mood: ↘ Recovery of mood after binge eating: BED < BN	" Binge eating in BED seems to be triggered by an immediate breakdown of emotion regulation." "Values for negative mood were higher and those for positive mood lower during binge days compared with non-binge days." "After binge eating, in contrast to findings from BN, we found a less pronounced and only slow recovery of mood after binge eating in BED."	- Only self-report measures (questionnaires, scales, or interviews) - Small sample size - Only treatment-seeking BED patients (no generalization possible to non-treatment-seeking BED) - Only women among participants
Görlach et al. [32] (Germany)	Participants with BMI < 30 (n = 124) Participants with BMI ≥ 30 (n = 190) Overeaters among them	Questionnaires / Scales	Emotion regulation Expressive suppression	Overeating frequency: Individuals with obesity > individuals without obesity	"[…] expressive suppression has a strong association with overeating in individuals with obesity." "Individuals with obesity reported more frequent overeating compared with individuals without obesity. " "[…] a moderating effect of BMI on the association of expressive suppression and overeating was found."	- Only self-report measures (questionnaires, scales, or interviews) - Data were collected via an internet survey: data may not be representative of the general population - Unequal balance of gender among participants
Svaldi et al., (2012) (Germany)	Women with BED (n = 25) Women with Anorexia Nervosa (n = 20) Women with Bulimia Nervosa (n = 18) Women with Major Depressive Disorder (n = 16) Female Healthy Controls (n = 42)	Questionnaires / Scales	Emotion regulation	For most ER variables: No significant differences between the ED groups Difficulties in ER: All clinical groups > HC Scores on the Difficulties in Emotion Regulation Scale (DERS) subscale “limited access to strategies”: BED < all other disorder groups	Participants with BED (like all other disorder groups) self-reported more emotion regulation problems than HC "Although differing from HC on this variable, participants with BED showed lower scores on the DERS subscale limited access to strategies than all other disorder groups."	- Only self-report measures (questionnaires, scales, or interviews) - The levels of depression were not considered - The sample sizes of the groups are not balanced, and some groups have very small sample sizes - Only women among participants
Kornacka et al. [20] (Poland)	Overweight / obese (n = 38) (Emotional Eaters among them) Control group (n = 50)	Questionnaires / Scales EMA (Ecological Momentary Assessment)	Rumination	Study 1: “[…] emotional eating mediates the link between rumination and uncontrolled eating or snacking, but only in healthy participants and not in the participants with overweight.” Study 2: “[…] when both momentary rumination and sad mood are entered into the model predicting momentary daily emotional eating, only rumination remains a significant predictor of emotional eating. This relationship is not modified by the fact that the participants are from healthy controls or the overweight/obese group.”	"[…] when both predictors (momentary rumination and affect) are included in the model predicting emotional eating, only rumination remains a significant predictor, suggesting that in daily functioning, rumination might be further explored as a mediator of the link between affect and emotional eating […]" "[…] the results of the two studies confirm the crucial role of ruminative thinking in the occurrence of emotional eating […]." "[…] in both healthy and participants with overweight rumination might lead to increased emotional eating, even when controlling for negative affect." "[…] emotional eating might be not caused directly by negative affect but it is enhanced by repetitive dwelling on the negative issue and negative affect itself." "The role of emotional eating in the link between rumination and uncontrolled eating is different in overweight vs. healthy individuals. Momentary rumination is linked to emotional eating."	- Only self-report measures (questionnaires, scales, or interviews) - Small sample sizes - Study may be underpowered for the computation of cross-sectional analyses - The gender of the participants was not mentioned
Schag et al. [29] (Germany)	Overweight or obese women with a diagnosis of BED or subthreshold BED (BED +) (= 25) Overweight or obese women without BED (BED-) (n = 26) Healthy, normal-weight women controls (NWC) (n = 25)	Free exploration paradigm Modified antisaccade paradigm Eye-tracking Questionnaires / Scales	Trait impulsivity Disinhibition, rash-spontaneous behaviour	Experiment 1: BED + participants gazed longer on food stimuli in comparison with BED- and NWC participants Experiment 2: “[…] BED + participants had more difficulties inhibiting saccades towards food and nonfood stimuli compared with both other groups in first saccades, and especially towards food stimuli in second saccades and concerning sequences of first and second saccades.” Performances in both experiments: BED- = NWC participants	"[…] food-related reward sensitivity and rash-spontaneous behaviour, as the two components of impulsivity, are increased in BED in comparison with weight-matched and normal-weight controls. This indicates that BED represents a neurobehavioural phenotype of obesity that is characterised by increased impulsivity."	- The rewarding stimuli are food-related only - Some participants had depressive symptoms - Only women among participants
Willem et al. [36] (France)	Obese adults (n = 120) (Emotional Eaters among them)	Questionnaires / Scales	Emotion dys-regulation	In Moderate Obesity: association emotion dys-regulation and more EE only through more anxiety In Severe Obesity: “[…] emotion dys-regulation was both directly and indirectly associated with more EE, but only through more depression in the latter.”	"Emotion dys-regulation, anxiety and depression have different impacts on emotional eating (EE) depending on obesity severity Emotion dys-regulation is only associated with more EE through more anxiety in moderate obesity Emotion dys-regulation is associated with more EE both directly and through more depression in severe obesity."	- Only self-report measures (questionnaires, scales, or interviews) - BMI was calculated from self-reported height and weight - Unequal balance of gender among participants
Gianini et al. [26] (USA)	Obese participants with BED (n = 326)	Questionnaires / Scales	Emotion regulation	Two DERS subscales (limited access to emotion regulation strategies and lack of emotional clarity) predicted emotional overeating in BED participants Two DERS subscales (nonacceptance of emotional responses and difficulties engaging in goal-directed behavior) predicted general eating pathology	"Emotion regulation may play a significant role in the maintenance of emotional overeating and eating pathology in obese adults with BED." "[…] limited access to emotion regulation strategies and lack of emotional clarity were the emotion regulation difficulties most strongly associated with emotional overeating." "Our results suggest that when obese individuals with BED experience negative affect they may lack effective strategies for managing these emotions." "Nonacceptance of emotional responses and difficulties engaging in goal-directed behavior were the emotion regulation difficulties most strongly associated with eating pathology."	- Only self-report measures (questionnaires, scales, or interviews) - Emotional Overeating was based upon recall of the past 28 days, which may be a too-long period - Only treatment-seeking BED patients (no possible generalization to non-treatment-seeking BED individuals) - Unequal balance of gender among participants
Racine & Horvath [33] (USA)	Women with Overeating only (n = 25) Women without pathological eating (No PE) (n = 137) Women with Loss of control only (LOC) (n = 32) Women with Objective binge episodes (OBEs) (n = 27)	Questionnaires / Scales	Emotion dys-regulation	Across all facets of emotion dysregulation: women with OBEs score > women No PE Women with OBEs: “[…] more problems with impulse control, less access to effective emotion regulation strategies, and greater overall emotion dysregulation than women with overeating only or LOC only.” Lack of emotional clarity: Women with OBEs = women with overeating	"The combination of overeating and LOC eating is associated with the greatest emotion dysregulation, but certain emotion regulation facets may differentially relate to overeating and LOC."	- Only self-report measures (questionnaires, scales, or interviews) - The sample sizes of the groups are not balanced, and some groups have very small sample sizes - Only women among participants
Sultson & Akkermann [19] (Estonia)	Women (n = 605) categorized in: Four-class model: Overweight women without Emotional Eating (n = 56) Obese women with Emotional Eating (n = 30) Normal weight women with Emotional Eating (n = 140) Normal weight women without Emotional Eating (n = 379) Five-class model**:** Obese women with Emotional Eating (n = 28) Normal weight women without Emotional Eating (n = 209) Normal weight women with Emotional Eating (n = 111) Normal weight women with Positive Emotional Eating (n = 196) Overweight women without Emotional Eating (n = 61)	Questionnaires / Scales	Emotion regulation	Level of ER difficulties and eating pathology: Obese and normal weight individuals with EE > other groups “Overweight individuals without EE showed moderate levels of eating pathology and low levels of ER difficulties […]” Normal weight individuals with positive EE: low levels of eating pathology, but moderate levels of ER difficulties	Four-profile model: "[…] the presence of high level of negative EE among profiles proved to be a particularly important indicator of ED psychopathology, whereas high BMI itself was not." Five-profile model: "[…] positive EE was associated with elevated levels of ER difficulties, suggesting that overeating in response to positive emotions might also include some features of emotion dysregulation." "Higher level of ER difficulties among obese and normal weight individuals with EE also lend further support for the assumption that emotion dysregulation might underlie EE."	- Only self-report measures (questionnaires, scales, or interviews) - The sample sizes of the groups are not balanced, and some groups have small sample sizes - Only women among participants
Wolz et al. [23] (Germany)	Healthy normal-weight women (n = 28) (Emotional Eating tested later)	EEG Go / No-go task Questionnaires / Scales	Inhibition	EE not correlated with habitual emotion suppression Go/No-go task: “[…] higher emotional eating scores were positively related to higher late positive potential (LPP) amplitudes in response to negative affective scenes.”	"[…] emotional eating tendencies might be related to increased neural reactivity specifically to negative stimuli." "[…] emotional eating was not related to general inhibitory control deficits, but was associated with higher behavioral inhibitory control difficulties only while suppressing negative emotions. Hence, the difficulty to inhibit behavioral responses while regulating negative emotions may contribute to disinhibited food intake while experiencing negative emotions." "The results of the current study suggest that people with emotional eating tendencies have response inhibition difficulties specifically when suppressing negative emotions […]" "Higher emotional eating scores were not related to higher scores in habitual use of emotion suppression." "No evidence was found supporting the hypothesis that emotional eating tendencies are related to generally lower inhibitory control capacities, but results showed preliminary evidence for an association between emotional eating and problems to inhibit behavioral responses specifically while trying to suppress negative emotions."	- EE scores may not be high enough among participants: the association between EE and suppression may be stronger in samples with higher mean EE scores - Measure of emotion suppression may not be sufficient - Only women among participants
Crockett et al. [3] (USA)	Undergraduate students (n = 552 including 334 women) (Emotional Eating tested later)	Questionnaires / Scales	Emotion regulation	Boredom proneness and emotion regulation predicted DEBQex scores	"In every model we tested, difficulties in emotion regulation predicted emotional eating." "Boredom proneness and emotional regulation were both strongly correlated with the emotional eating variables."	- Only self-report measures (questionnaires, scales, or interviews) - All participants were undergraduate students (cannot generalize to the general population)
Svaldi et al. [39] (Germany)	Women with BED (n = 31) Group without BED / No-BED (n = 29)	Questionnaires / Scales Stop Signal Task (SST)	Behavioral inhibition	Stop signal reaction time (SSRT): BED > No-BED Difficulty inhibiting responses elicited by food stimuli: BED > No-BED “The deficits in behavioral response inhibition were also found to be related to the severity of reported symptoms.”	"The stop signal reaction time (SSRT) was found to be increased in BED participants compared with the No-BED group, indicative of a deficit in response stopping in BED." "Of note, both previous and current findings suggest that response inhibition at different stages of the response process may be impaired in BED." "[…] the BED group experienced more difficulty inhibiting a response elicited by food stimuli than by neutral stimuli." "In conclusion, the present study supports a general deficit in late stage response inhibition in BED relative to obese No-BED group, particularly when the response is elicited in the context of food stimuli. Additionally, the magnitude of the inhibitory deficit was found to be related to the reported severity of eating pathology."	- Food consumption before the study was not assessed - Only women among participants
Wood et al. [24] (USA)	Healthy adults (n = 20) (Emotional Eaters among them)	Questionnaires / Scales fMRI (functional magnetic resonance imaging) Go / No-go task	Self-control (inhibition)	Emotional eating: Positive associations with insula and dorsolateral prefrontal cortex activation in response to high-calorie versus low-calorie foods Positive associations with dorsolateral prefrontal cortex activation in response to approach versus inhibition towards high-calorie foods	"[…] our results demonstrate an increase in activation across brain regions related to self-control and urges in response to high-calorie food associated with both emotional eating and routine restraint."	- Go / No-go task only performed with food-related stimuli and no other rewarding stimuli - Compensatory restraint and external eating may not induce differential brain activation in response to a food-related Go / No-go task - Statistical power may be not powerful enough to detect an association between the reward circuitry and the external eating / compensatory restrain
Mobbs et al. [38] (Switzerland)	Obese patients with BED (n = 16) Obese patients without BED (n = 16) Normal-weight controls (n = 16)	Questionnaires / Scales Mental flexibility task	Inhibition	Errors rate and omissions rate: All obese patients > controls Obese patients with BED > obese patients without BED	“Concerning our second objective, namely to compare obese patients with and without binge eating disorder, the pattern of results (increased errors for all types of stimuli in the whole task, increased omissions for all types of stimuli in the food section of the task and absence of performance improvement in the food section for obese patients with binge eating disorder) suggests that obese patients with binge eating disorder have a more severe global impairment of inhibition and more difficulty focusing their attention. This result raises the possibility that there is a continuum of increasing inhibition and cognitive problems with increasingly disordered eating among obese patients.” “[…] the difference between obese persons with binge eating disorder and obese persons without binge eating disorder suggests that the former have a more severe fundamental inhibition problem.” “[…] these cognitive deficits are more severe in obese patients with binge eating disorder, which indicates that there is a continuum of increasing inhibition and cognitive problems with increasingly disordered eating.”	- The shifting rule may be too easy to observe differences between obese patients and normal-weight controls - The shifting task does not allow to assess the nature of the inhibition deficit - Unequal balance of gender among participants
Mole et al. (2015) (UK)	EtOH—abstinent alcohol-dependent subjects (n = 30) HV-EtOH—healthy volunteers abstinent alcohol-dependent subjects (n = 30) Obese subjects with BED (n = 30) HV—healthy volunteers (n = 30) Obese controls (n = 30) HV—healthy volunteers (n = 30)	Questionnaires / Scales Delay discounting task (DTT) Stop signal task (SST) Information sampling task (IST)	Impulsivity	Stop signal task: "In BED subjects, there were no significant differences in GoRT […] compared to HV.” (GoRT = Go-trial Reaction Time) “Obese subjects without BED compared to those with BED also had greater impairments in motor response inhibition […]” Information sampling task: "There were no significant differences between HV and BED subjects […]” Delay discounting task: "All three groups had greater delay discounting relative to healthy volunteers.”	"Our findings are compatible with both a conceptualization of BED as an extreme neurobehavioural subtype of obesity and BED having similarities with other behavioural and substance addictions." “Unexpectedly, obese subjects without BED showed greater impulsivity than obese subjects with BED.”	- Income was not taken into account for the DDT - Rather small sample sizes
Smith et al. (2020) (USA)	Women with BED (n = 29) Women with Bulimia Nervosa (BN) (n = 9) Woman with AN-BP (anorexia nervosa binge-purge type) (n = 1) Woman with and Other Specified Feeding or Eating Disorder (OSFED, subthreshold BED presentation) (n = 1) "Participants were grouped based on their DSM-5 ED diagnoses, such that those with AN-BP or BN were categorized in the “compensatory behavior” group, and those with BED or subthreshold BED were categorized in the “no compensatory behavior” group."	Questionnaires / Scales EMA (Ecological Momentary Assessment) Ambulatory Go / No-go task	Inhibitory control Affect	See the “Outcomes” column	"[…] there were no significant independent or interactive effects of momentary negative affect and daily inhibitory control in predicting binge eating among individuals with BED or subthreshold BED."	- The Go / No-go task may not be challenging enough - The sample sizes of the groups are not balanced, and some groups have very small sample sizes - Only women among participants
Leehr et al. [30] (Germany)	Obese women with BED (n = 21) Obese controls (women) without BED (n = 23) Normal-weight healthy controls (women) (NWC) (n = 25)	Questionnaires / Scales Eye-tracking Antisaccade task Genotyping	Impulsivity (trait and behavioural) Inhibitory control	See the “Outcomes” column	"[…] the BED + sample showed higher trait and behavioural impulsivity. Furthermore, within the BED + group, COMT Met/Met homozygous individuals showed stronger deficits in inhibitory control." "COMT Met/Met homozygous individuals with BED might represent a specific group in the BED spectrum, which shows a higher behavioural impulsivity." "[…] our results hint towards an interaction of the COMT Met/Met homozygote genotype with increased behavioural impulsivity and suggest that this might play a role in binge eating mechanisms in BED."	- Behavioral impulsivity was only measured with high caloric food-related stimuli - Small sample sizes - Only women among participants
Eneva et al. (2017) (USA)	Overweight women with BED (OW-BED) (n = 32) Normal-weight women with BED (NW-BED) (n = 23) Overweight women without BED (OW-HC) (n = 48) Normal-weight women without BED (NW-HC) (n = 29)	Questionnaires / Scales Set of tests that assesses higher-level cognitive functions Clinical interviews	Inhibitory control	“NW-BED individuals demonstrated significantly greater inhibition as measured by the Flanker Inhibitory Control task than all other groups.”	"In contrast to a past study that showed that obese individuals with BED did better on motor inhibitory tasks than obese HCs (Mole et al., 2015), we did not observe better performance in our OW-BED group, only in our NW-BED group. It is possible that the relatively higher inhibitory control observed in NW-BED relative to all other groups serves as a protective factor, preventing weight gain." “Thus, our findings suggest that EF of participants in the NW range, even with a diagnosis of BED, is generally characterized by better psychomotor performance than OW, as captured by multiple EF tasks.” "Replication of the finding that normal-weight BED is associated with enhanced inhibitory control is needed."	- Only women among participants - The sample sizes of the groups are not balanced, and some groups have small sample sizes
Balodis et al. (2013) (USA)	Obese BED individuals (n = 11) Non-BED obese individuals (OB) (n = 13) Lean comparison group (LC) (n = 11)	Questionnaires / Scales fMRI Stroop task	Inhibitory control	“[…] the BED group showed diminished activity in the ventromedial prefrontal cortex (vmPFC), inferior frontal gyrus (IFG), and insula during Stroop performance.”	"Relative to the OB and LC groups, activity in the BED group was differentiated by relative hypoactivity in brain areas involved in self-regulation and impulse control." "[…] the BED group demonstrated diminished activity in frontal regions subserving inhibitory control, including the vmPFC and the IFG. In addition, diminished activity was also noted in the insula, in superior and middle temporal areas, as well as in the middle occipital gyrus." "[…] BED individuals’ diminished ability to recruit impulse-control-related brain regions appears associated with impaired dietary restraint. The observed differences in neural correlates of inhibitory processing in BED relative to OB and LC groups suggest distinct neurobiological contributions to binge eating as a subgroup of obese individuals."	- The Stroop task does not only involve cognitive control, but also many other cognitive processes - No Stroop behavioral measures during fMRI scanning - Low contrast map threshold (p < .05 for group comparison) - Small sample sizes - The average age is much higher in the BED group than in the other groups - Only treatment-seeking BED patients (no generalization possible to non-treatment-seeking BED individuals)
Brockmeyer et al. (2014) (Germany)	Women with AN-R (anorexia nervosa-restricting type) (n = 35) Women with AN-BP (anorexia nervosa–binge/purge type) (n = 22) Women with BN (bulimia nervosa) (n = 34) Women with BED (binge-eating disorder) (n = 29) Normal-weight controls (NWC) (n = 60) Over-weight controls (OWC) (n = 29)	Questionnaires / Scales	Emotion regulation	ER difficulties (experience and differentiation of emotions + attenuation and modulation of emotions): all ED subtypes > controls Some ER domains difficulties: Other ED subtypes > BED > controls Impulse control: BN = BED = AN-R ER difficulties in impulse control: AN-BP > BED > BN	"[…] all ED subtypes reported significantly more ER difficulties than healthy controls in the domains of both experience and differentiation of emotions as well as attenuation and modulation of emotions." "[…] BED reported less problems than other ED subtypes regarding some ER domains, albeit still differing from healthy controls." "However, that BN and BED did not differ from AN-R regarding impulse control is rather surprising." "Our fourth hypothesis (impulse control difficulties: AN-BP, BN > BED) also received only partial support since AN-BP but not BN exceeded BED regarding ER difficulties with impulse control." "[…] BN did not show greater ER difficulties in impulse control than BED in the present study, which is rather surprising, given that BN used to show more problems than BED regarding different facets of impulsivity." "[…] self-reported general impulse control difficulties in the context of ER seem to measure a different aspect of impulsivity than neuropsychological tests which BN and BED may share with each other." "The findings underscore the relevance of ER difficulties in ED and support the trans-diagnostic view of ER difficulties being present across the whole spectrum of ED. In addition, the present results suggest that certain domains of ER may be linked more closely to certain ED subtypes than to others."	- Only self-report measures (questionnaires, scales, or interviews) - The sample sizes of the groups are not balanced, and some groups have rather small sample sizes - The treatment status was not considered - Only the DERS was used to measure impulsivity (impulsivity subtypes could have been missed) - Only women among participants
Ruscitti et al. (2016) (USA)	EDNOS (Eating Disorder, Not Otherwise Specified) (n = 120) Anorexia Nervosa (n = 29) Bulimia Nervosa (n = 22) Binge Eating Disorder (n = 20)	Questionnaires / Scales	Emotion regulation	Significant differences in ER: BED and EDNOS > psychiatric patients without EDs Difficulties in Limited Access to Emotion Regulation Strategies: BED > EDNOS	"[…] individuals with EDs have greater ER difficulties in most domains of ER and that those with BED and EDNOS demonstrate the most significant differences in ER as compared to psychiatric patients without EDs." "[…] it was found that ED subtypes typically did not differ in terms of specific difficulties in ER. One exception emerged indicating that individuals with BED demonstrated significantly greater difficulty on the Limited Access to Emotion Regulation Strategies subscale as compared to those with EDNOS."	- Only self-report measures (questionnaires, scales, or interviews) - The sample sizes of the groups are not balanced, and a very small sample of BED compared to EDNOS - No healthy weight-matched control subjects - ED within the EDNOS group can greatly vary - Unequal balance of gender among participants
Deroost & Cserjési [21] (Belgium)	Low Emotional eaters group (n = 18) High Emotional eaters group (n = 23)	Questionnaires / Scales Exogenous Cueing Task (ECT)	Emotion regulation Attentional avoidance	Attentional avoidance of emotional faces (particularly negative faces): High EM group significant* Low EM group not significant Levels of avoidance coping: High EM group > Low EM group	"[…] these results indicate that the high EM group directed their attentional focus in a way that allowed them to avoid elaborative attentional processing of emotional stimuli." "Thus, both the ECT results and the self-reported data suggest that people with a high degree of EM use avoidance as a primary coping strategy." "Avoidance coping also significantly predicted the level of EM." "[…] overall, our results are in agreement with the hypothesis that EM acts as a maladaptive coping mechanism to avoid emotional distress […]."	- Small sample sizes - Unequal balance of gender among participants, especially in the High EM group
Aloi et al. [31] (Italy)	BED patients (n = 155)	Questionnaires / Scales Network analysis (NA)	Emotion dysregulation Impulse control	See the “Outcomes” column	"[…] according to the present NA findings, impaired self-monitoring metacognition and difficulties in impulse control are the central nodes in the psychopathological network of BED, whereas eating symptoms seem to be marginal."	- Only self-report measures (questionnaires, scales, or interviews) - Small sample size compared to other studies that used NA in BED - Unequal balance of gender among participants
Stapleton & Whitehead [18] (Australia)	Australian adults (n = 223) (Emotional Eating tested later)	Questionnaires / Scales	Emotion regulation Impulsivity	Difficulties with ER, impulsivity and sensitivity toward rewards: dysfunctional emotional eaters > non-dysfunctional emotional eaters The best at predicting emotional eating behavior: 1. ER difficulties, 2. impulsivity, 3. sensitivity to reward, 4. sensitivity to punishment	"Emotional eaters significantly differed from those who did not engage in dysfunctional levels of emotional eating in terms of their emotion regulation, impulsivity, and sensitivity towards reward, and difficulties in emotion regulation predicted emotional eating." "It was proposed that emotion regulation difficulties would contribute the most towards predicting emotional eating behaviour, followed by impulsivity, sensitivity to reward, and then sensitivity to punishment." "Higher levels of emotion regulation difficulties were associated with emotional eating […]." "Emotion regulation difficulties was the greatest predictor of emotional eating, suggesting that individuals who have difficulty regulating their emotions are more likely to engage in emotional eating behaviour."	- Only self-report measures (questionnaires, scales, or interviews) - Non-clinical population: findings may not be directly applicable to ED patients - Unequal balance of gender among participants
Wang et al. [37] (USA)	Obese participants with BED (n = 237)	Questionnaires / Scales	Rumination (brooding and reflective)	“Hierarchical multiple regressions indicated that rumination was associated with eating-disorder psychopathology and weight-bias internalization above and beyond the influence of overvaluation of shape/weight.”	"Findings suggest that, among patients with BED/obesity, rumination is an important cognitive process associated with severity of eating-disorder psychopathology even after accounting for overvaluation of shape/weight. Patients with greater rumination might be more likely to dwell on weight-based discrimination experiences and internalize these negative attitudes."	- Only self-report measures (questionnaires, scales, or interviews) - Only treatment-seeking BED patients (no possible generalization to non-treatment-seeking BED individuals) - Unequal balance of gender among participants

*EE* Emotional Eating, *BED* Binge-Eating Disorder, *ER* Emotion Regulation, *EEG* Electroencephalography, *MEG* Magnetoencephalography, *NWC* Normal Weight Controls, *FST* Food Stroop Task, *BMI* Body Mass Index, *BIS-11* Barratt Impulsiveness Scale, *EMA* Ecological Momentary Assessment, *BN* Bulimia Nervosa, *HC* Healthy Controls, *ED* Eating Disorders, *DERS* Difficulties in Emotion Regulation Scale, *BED* + People living with BED (or subthreshold BED, depending on the studies), *BED-* People living without BED (or subthreshold BED, depending on the studies), *LOC* Loss of Control, *OBE* Objective Binge Episode, *LPP* Late Positive Potential, *SST* Stop Signal Task, *SSRT* Stop Signal Reaction Time, *fMRI* functional Magnetic Resonance Imaging, *HV* Healthy Volunteers, *GoRT* Go-trial Reaction Time, *NW-BED* Normal-weight people living with BED, *OW-BED* Overweight people living with BED, *EF* Executive Functioning, *OB* Non-BED obese individuals, *LC* Lean comparison, *vmPFC* ventromedial prefrontal cortex, *IFG* inferior frontal gyrus, *AN-R* Anorexia Nervosa-Restricting type, *AN-BP* Anorexia Nervosa–Binge/Purge type, *EDNOS* Eating Disorder, Not Otherwise Specified, *EM* Emotional Eaters (depending on the studies), *ECT* Exogenous Cueing Task, *NA* Network Analysis

### Critical analysis

Two authors (M. A. and P.-E. B.) listed the possible limitations of each study. The limitations identified by both authors were retained, and the others were either eliminated or retained after discussion. A third author (S. C.) checked this list, and her comments were considered. The limitations are summarized in the "Limitations" column of Table 1 and are discussed in Sect. "Critical analysis".

### Synthesis of results

Data were analyzed qualitatively. We first grouped the studies by the types of eating behaviors (emotional eating (EE), overeating (OE), and binge-eating disorder (BED)). Then, we addressed each of the topics formulated in our questions/hypotheses.

## Results

### Selection of sources of evidence

The source search in the electronic bibliographic databases retrieved 2596 records (2130 on PubMed/Medline and 466 on PsycINFO) (see Fig. 2). After removing 301 duplicate records, 2295 records were screened in the first screening step. During this first screening step, for each of the 2295 articles, the inclusion criteria described in the *Eligibility criteria* section of the *Methods* section were applied to both titles and abstracts, resulting in 110 records to be assessed for eligibility in the next step. Thus, in the second screening step (eligibility), for each of the 110 articles, the same inclusion criteria were applied to the entire article (i.e., a complete reading of the article). At the end of this second stage, 32 studies were selected to be included in the review. Table 1 shows the main characteristics of the 32 studies included in the scoping review, according to the variables described in Sect. "Data charting process and data items".

Regarding the types of populations (cf. PICOS criteria), 9 studies out of 32 focused on emotional eating (EE), 21 studies out of 32 focused on binge-eating disorder (BED), and 2 out of 32 focused on overeating (OE). Regarding the types of intervention/exposure (cf. PICOS criteria), namely, ER and inhibition/impulsivity, 19 studies out of 32 focused on emotion regulation (ER), 18 studies out of 32 focused on inhibition/impulsivity, and 5 out of 32 focused on both ER and inhibition/impulsivity. More than a third of the included studies (11 studies out of 32, ≈ 34%) were conducted in Germany. Moreover, more than half of the studies (17 studies out of 32, ≈ 53%) were conducted in Germany or in countries bordering Germany (i.e., France, Switzerland, Belgium and Poland).

### Summary of findings

#### Emotional eating and emotion regulation

Studies confirm the existence of a link between ER and EE, including the fact that ER difficulties predict EE. For example, Stapleton and Whitehead [18] highlighted that “Emotion regulation difficulties was the greatest predictor of emotional eating, suggesting that individuals who have difficulty regulating their emotions are more likely to engage in emotional eating behavior”. Similarly, Crockett et al. [3] concluded that “In every model we tested, difficulties in emotion regulation predicted emotional eating”. Sultson and Akkermann [19] concluded that "Higher level of ER difficulties among obese and normal weight individuals with EE also lend further support for the assumption that emotion dysregulation might underlie EE". Kornacka et al. [20] highlighted the “[…] crucial role of ruminative thinking in the occurrence of emotional eating […]”. Regarding avoidance, Deroost and Cserjési [21] showed “[…] that people with a high degree of EM use avoidance as a primary coping strategy" and added that "avoidance coping also significantly predicted the level of EM” (EM = emotional eating).

Future studies focusing on EE and ER should separately test other specific types of emotional eating (e.g., EE in response to depression, to anxiety…). Indeed, Braden et al. [22] explained that “exploratory analyses suggest possible unique relationships between types of emotional eating and specific facets of emotion regulation”. The authors added that “[…] findings suggest that certain emotion regulation strategies may be more closely linked to various types of emotional eating”.

#### Emotional eating and inhibition

The studies included in this scoping review dealing with EE and inhibition/impulsivity confirmed the existence of an association between EE and some inhibition difficulties and impulsivity. For example, Wolz et al. [23] showed that “[…] emotional eating was not related to general inhibitory control deficits, but was associated with higher behavioral inhibitory control difficulties only while suppressing negative emotions. They added that “[…] the difficulty to inhibit behavioral responses while regulating negative emotions may contribute to disinhibited food intake while experiencing negative emotions”. Stapleton and Whitehead [18] revealed that emotional eating was related to high impulsivity and that impulsivity was the second greatest predictor of EE after emotion regulation difficulties. Regarding self-control, Wood et al. [24] showed “[…] an increase in activation across brain regions related to self-control and urges in response to high-calorie food associated with both emotional eating and routine restraint". Taken together, these findings confirm that emotional eaters are prone to inhibition impairments. Moreover, Wolz et al. [23] suggest that deficits in inhibition only appear when participants are regulating their emotions, highlighting an interesting link between ER and inhibition in EE.

#### BED and emotion regulation

Concerning BED and emotion regulation, most of the studies confirm the ER difficulties in BED. Leehr et al. [25] showed that individuals with BED have lower ER capacities. Limited access to ER strategies is also one of the ER difficulties met in BED [26, 27], as well as nonacceptance of emotional responses [27] and lack of emotional clarity [26, 27].

#### BED and inhibition

Overall, studies focusing on BED indicated a deficit in inhibition and increased impulsivity. Leehr et al. [25] concluded that “Overall, results support the assumption of inhibitory control deficiencies in BED on a behavioral level”. Grant and Chamberlain [28] underlined that “Binge-eating disorder was associated with impaired response inhibition and executive planning”. Schag et al. [29] said that “[…] BED represents a neurobehavioural phenotype of obesity that is characterized by increased impulsivity”, and Leehr et al. [30] showed that “the BED + sample showed higher trait and behavioural impulsivity”. Moreover, according to Aloi et al. [31], “[…] impaired self-monitoring metacognition and difficulties in impulse control are the central nodes in the psychopathological network of BED […]”.

#### Overeating and emotion regulation

One of the objectives of this scoping review was to clarify the ill-defined concept of *overeating*. In the eating disorders literature, overeating sometimes refers to a symptom of an eating disorder or as an eating behavior or is sometimes used as a synonym for emotional eating or binge eating.

Unfortunately, only two of the studies included in this scoping review focused on overeating [32, 33], so we could not address this specific question. Nevertheless, similar to EE and BED, those studies highlighted the links between overeating and emotion regulation.

#### Positive emotions and emotional eating

We questioned the possibility of positive emotions causing emotional eating episodes associated with emotion regulation and/or inhibition difficulties (in the same way as negative emotions). Based on the studies included in our review, opinions differ regarding this point. Indeed, while an article highlights that “[…] positive EE was associated with elevated levels of ER difficulties, suggesting that overeating in response to positive emotions might also include some features of emotion dysregulation” [19], another article concludes, on the contrary, that “[…] eating in response to positive emotions was not significantly related to poorer psychological well-being, greater eating disorder symptoms, or emotion dysregulation” [22]. Since there is yet no consensus on the subject, further research on emotional eating needs to be conducted to separately test and dissociate positive and negative emotions.

#### Positive emotions and BED

We wondered about positive emotions as a possible cause of emotional eating episodes associated with emotion regulation and/or inhibition difficulties. We checked whether the BED studies included in this scoping review addressed the question of positive emotions/affect/mood. Loeber et al. [34] showed that “[…] restrained eating and mood are factors that moderate response inhibition to food-associated stimuli in obese patients with BED” and that “[…] apart from negative mood, positive mood might as well be a trigger for loss of control over eating behaviour”.

Finally, it is worth mentioning that one study showed that negative and positive mood levels are different during binge days, with an increasing negative mood and a decreasing positive mood at the first binge-eating episode (see Munsch et al. [35]).

#### Emotional eating and weight profiles

Studies included in this scoping review tended to show that the relationships between EE and emotion dysregulation (and anxiety, depression, and rumination) might be different according to the weight profile (i.e., normal weight, overweight, and with moderate or severe obesity) (see, for example, Willem et al. [36] or Kornacka et al. [20]). Willem et al. [36] highlighted that “emotion dysregulation, anxiety and depression have different impacts on emotional eating (EE) depending on obesity severity", while Kornacka et al. [20] underlined that “the role of emotional eating in the link between rumination and uncontrolled eating is different in overweight vs. healthy individuals”.

#### Emotional eating, BED and rumination

According to three of the studies included in this scoping review, rumination, a maladaptive emotion regulation strategy, is encountered in both EE and BED. Indeed, in EE, Kornacka et al. [20] highlight that rumination is a predictor of EE (“[…] the results of the two studies confirm the crucial role of ruminative thinking in the occurrence of emotional eating […]”. Similarly, people with BED are more inclined than healthy people to use rumination as a negative emotion regulation strategy [27]. Wang et al. [37] also highlighted that “[…] rumination is an important cognitive process associated with severity of eating-disorder psychopathology”.

#### The idea of a possible continuum

We hypothesized that there would be a continuum between EE (nonpathological eating behavior) and BED (pathological eating behavior). Three studies focusing on BED mentioned this idea of a continuum in the severity of eating disorders. Leehr et al. [25] stated that “From a clinical perspective eating behavior of the three groups can be seen on a continuum from normal eating behavior, to overeating, to binge eating”. Mobbs et al. [38] highlighted that “[…] these cognitive deficits are more severe in obese patients with binge eating disorder, which indicates that there is a continuum of increasing inhibition and cognitive problems with increasingly disordered eating”. Moreover, Svaldi et al. [39] underlined that “[…] the magnitude of the inhibitory deficit was found to be related to the reported severity of eating pathology”, which is compatible with the idea of a continuum.

However, none of the reviewed studies directly compared EE to BED regarding ER or inhibition performances, neither in a longitudinal nor cross-sectional design. Thus, a gap can clearly be identified in this specific field since there is a complete lack of experimental data about an increased severity in ER and inhibition deficit between EE and BED.

## Critical analysis

We identified some limitations between studies, and some of them were quite redundant in our corpus. First, half of the included studies recorded only self-reported data using scales, questionnaires, or interviews. These declarative measures often suffer from memory bias or social desirability concerns [40]. Moreover, these measures are often carried out for a particular purpose, and this purpose may differ from study to study, depending on the research question being asked [40]. Strikingly, 88.9% of papers addressing ER gathered only self-report measures (but only 11.8% for inhibition). Thus, there is a lack of experimental data to address the issue of ER in BED and EE.

Second, 43.8% of the articles with self-report measures only appeared to have rather small sample sizes and/or unbalanced groups and were therefore underpowered. Sample size is a critical issue for quantitative analysis. This sample size must be large enough to achieve the appropriate level of measurement precision. [41].

Third, most of the participants enrolled in these studies were women, compromising the generalizability to the global population (81.3% of studies had only women participants or an unbalanced sex ratio toward women). Eating disorders are more frequent among women, and for BED, the ratio varies between 1:2 and 1:6 [42]. Thus, while the lack of men in BED studies is understandable, future studies should consider recruiting more men to properly balance the experimental groups.

Finally, a recurring limitation emphasized by many authors of the included studies is that their research was cross-sectional. Indeed, given the short duration of these types of studies, it was impossible to reveal some causal links between different phenomena (e.g., between BED and impulsivity). However, in our opinion, this is not a limitation per se, as cross-sectional and longitudinal studies are two very different types of research. Therefore, we did not report this limitation in Table 1.

## Discussion

The main objectives of this scoping review were to explore the idea of a continuum between EE and BED as well as explore the idea of a gradation in emotion regulation and inhibition deficits along this continuum. This hypothesis is supported by some authors and is widely discussed in Davis [14]. He developed the concept of an “eating continuum”, ranging from homeostatic eating (energy balance) to food addiction, with different levels of “overeating”, including BED-like symptoms and diagnosed BED. It should also be noted that this idea of a continuum is shared by many physicians in their daily clinical practice and that this idea needs to be verified.

The most striking result of our scoping review is that there are strong similarities between EE and BED, with emotional eaters and BED patients sharing the same difficulties in emotion regulation and inhibition. Some of the included studies seem to be compatible with the idea of a gradation of ER and inhibition deficits following this continuum. For instance, Mobbs et al.’s [38] conclusions strengthened the idea of a continuum of inhibition impairment, with BED patients living with obesity having more difficulties inhibiting their responses compared to controls living with obesity. Indeed, the authors concluded that “[…] these cognitive deficits are more severe in obese patients with binge eating disorder, which indicates that there is a continuum of increasing inhibition and cognitive problems with increasingly disordered eating”. Concerning EE, the results of Sultson and Akkermann [19] showed that participants with EE have more binge eating behaviors than participants without EE but do not meet all the DSM-5 criteria to be diagnosed with BED. These results suggest that EE could lead to BED and thus support the idea of a continuum. It is, however, crucial to remember that none of the articles included in this review directly compared EE and BED in the same study, neither in a longitudinal nor cross-sectional design. To ascertain the existence of a continuum between EE and BED, the increased severity of ER and inhibition deficits between EE and BED still need to be proven. One of the main goals of this scoping review was also to identify knowledge gaps, and indeed, we found a gap in the literature regarding the increased severity in ER and inhibition impairments from EE to BED. Such a lack of experimental work is truly surprising given the feelings shared by many caregivers in the field of eating disorders as well as the thoughts shared by some authors [14, 25, 38, 39].

Among the thirty-two articles reviewed, only one focused on the relationship between ER and inhibition in EE. Indeed, Wolz et al. [23] showed that EE was associated with higher behavioral inhibitory control difficulties only while participants were suppressing negative emotions. This outcome should be taken into account in further studies, since ER and inhibition deficits are often studied separately [7, 43, 44]. Indeed, the direct relationship between ER and inhibition remains poorly studied in BED, as well as in EE, but is an important question to explore the idea of a continuum.

The third objective of this scoping review was to address the ill-defined concept of overeating. Unfortunately, only two studies focused on overeating [32, 33], and it is thus difficult to clearly define this concept. For both authors, overeating is not an eating disorder per se since participants were healthy volunteers with no prior diagnosis of an eating disorder. However, in both studies, overeating is measured with questionnaires widely used in medical contexts to assess eating disorders, such as the Eating Disorder Examination-Questionnaire (EDE-Q) or the Binge Eating Scale (BES). Thus, overeating may be seen as pathological eating. Moreover, Racine and Horvath [33] used the Eating Disorder Diagnostic Scale and the Questionnaire on Eating and Weight Patterns-5 (QEWP-5) to determine experimental groups. Women included in the “overeating” group reported consuming an “unusually large amount of food unaccompanied by loss of control over the past 3 months” on both questionnaires. Thus, this inclusion criterion could be a suitable definition of the concept of overeating, but it must be emphasized that there is too little information to properly define this concept.

The fourth aim of this review was to determine whether positive emotions could trigger emotional eating or binge eating episodes associated with emotion regulation and/or inhibition difficulties. Most of the studies only measured EE and binge eating episodes in response to negative emotions. However, few articles specifically focused on positive mood or emotions. Due to a lack of consensus among studies, it was impossible to strongly conclude that positive emotions can affect eating behaviors. Indeed, some data support this idea [19, 34], and others are less affirmative [22, 35].

Last, concerning the weight profile, it was not one of the aims of this scoping review, but our results showed that emotion regulation deficits were more severe in obese participants than in normal weight or overweight volunteers. Thus, the weight profile seems to be an important parameter when addressing the question of an increased severity in ER deficits between EE and BED.

## Limitations

This scoping review presents some limitations. First, regarding the selection phase, not all relevant studies may have been indexed in the two searched databases (PsycINFO and PubMed/Medline). Second, the examination was based on a list of terms describing emotional eating, binge-eating disorder, emotion regulation and inhibition. The possibility that additional articles would have been identified by adding other terms cannot be completely excluded, although the search was intended to be as extensive as possible. Third, a possible limitation of our scoping review is that we did not mention explicitly in our search equation the terms “positive emotions”. Indeed, given that one of our questions was about the possibility that positive emotions can, like negative emotions, trigger emotional eating episodes, we could have perhaps included it in our search equation. Nevertheless, given that we used the inclusive terms “Emotional Regulation”[Mesh]” and “Emotion regulation”, it is likely that we did not miss some interesting records focusing on positive emotions. Finally, in this review, only studies in French or English were included, which did not allow us to be exhaustive in our conclusions.

## Conclusion and further directions

In conclusion, this scoping review fully confirmed the presence of inhibition and emotion regulation deficits in both EE and BED, showing strong similarities between these two eating behaviors. However, the lack of experimental data coming from direct comparisons between EE and BED did not make it possible either to confirm the existence or the absence of a possible continuum between EE and BED or an increased severity in ER and inhibition deficits between EE and BED. Thus, this scoping review helped to identify a knowledge gap, and the question of the existence of a continuum still needs to be addressed in further research.

If such a continuum exists, we think it could greatly impact the clinical care of eating disorders. Indeed, if EE can become BED, early care of emotional eaters becomes essential, and early diagnoses could be made. Additionally, prevention could be improved in emotional eaters and even in the general population to avoid progression to an eating disorder (i.e., subthreshold BED and BED) and could also reduce the risk of developing obesity and its comorbidities often associated with BED. Given the variety of symptoms (psychological and physical), monitoring of emotional eaters could be performed by a multidisciplinary medical team, especially for children and adolescents.

The existence of a continuum between EE and BED could also have implications for eating disorder research. In our view, this could lead to further research to develop more specific screening instruments, such as scales and questionnaires. Such instruments might indeed be helpful to classify emotional eater participants into more relevant experimental groups that take into account the severity of EE. To go even further, one could imagine a new scale that would assess the level of eating behaviors across the entire continuum. Moreover, regarding data analysis, data could be analyzed in a discrete way in addition to group comparisons between EE and BED. Last, if such a continuum was verified, it could guide the focus on future research, especially studies on the etiology of BED, and help to better define the concept of “emotional overeating”.

Moreover, to test the idea of a continuum from a different angle, it could be interesting to see if there is an evolution of some other markers between EE and BED, such as biomarkers. Some of them are well known in BED but remain rather poorly studied in EE. Several fMRI studies have shown that brain activation patterns are different in BED patients, especially in the reward system, which explains why this eating disorder is often associated with food addiction [14]. For example, the ventral striatum and the medial prefrontal cortex seem to be underactivated during a rewarding task. Moreover, the ventral putamen, orbitofrontal cortex, amygdala, and insula respond less in BED patients than in controls [8]. EEG studies have also provided a valuable understanding of neurophysiological markers. In their narrative review, Berchio et al. [45] found that behavioral traits of BED and bulimia nervosa, such as loss of control over eating and emotional eating, are associated with an increased attentional reactivity (P300 wave) to visual food stimuli. Finally, animal studies allow us to better understand the functioning of some molecules. For example, the role of dopamine, oxytocin, and opiate in eating disorders is well understood [46], and this could be an interesting focus to measure the gradation between EE and BED.

## Data Availability

Two electronic bibliographic databases, PubMed/Medline and PsycINFO, were searched to identify references related to the scoping review topic. The search focused on articles published between January 2009 and January 2022. Original records (before screening) can be found using the search equation that was used in both databases: ("Binge-Eating Disorder"[Mesh] OR BED OR Binge eater OR Emotional Eating OR Emotional Overeating OR Overeater OR Emotional eater OR Overeating) AND ("Emotional Regulation"[Mesh] OR Emotion regulation OR Reappraisal OR Rumination OR Attentional deployment OR Mood regulation OR "Inhibition, Psychological"[Mesh] OR Inhibitory control). The 32 articles included after the screening steps are listed in the References section.

## Abbreviations

EE: Emotional eating
BED: Binge-eating disorder
ER: Emotion regulation
PRISMA-ScR: Preferred reporting items for systematic reviews and meta-analyses extension for scoping reviews
PICOS criteria: Populations, interventions, comparisons, outcomes, studies
DSM-IV-TR, or DSM-5: Diagnostic and statistical manual of mental disorders Text Revision, or 5th edition
EEG: Electroencephalography
MEG: Magnetoencephalography
NWC: Normal weight controls
FST: Food stroop task
BMI: Body mass index
BIS-11: Barratt impulsiveness scale
EMA: Ecological momentary assessment
BN: Bulimia nervosa
HC: Healthy controls
ED: Eating disorders
DERS: Difficulties in emotion regulation scale
BED +: People living with BED (or subthreshold BED, depending on the studies)
BED-: People living without BED (or subthreshold BED, depending on the studies)
LOC: Loss of control
OBE: Objective binge episode
LPP: Late positive potential
SST: Stop signal task
SSRT: Stop signal reaction time
fMRI: Functional magnetic resonance imaging
HV: Healthy volunteers
GoRT: Go-trial reaction time
NW-BED: Normal-weight people living with BED
OW-BED: Overweight people living with BED
EF: Executive functioning
OB: Non-BED obese individuals
LC: Lean comparison
vmPFC: Ventromedial prefrontal cortex
IFG: Inferior frontal gyrus
AN-R: Anorexia nervosa-restricting type
AN-BP: Anorexia nervosa–binge/purge type
EDNOS: Eating disorder, not otherwise specified
EM: Emotional eating/eaters (depending on the studies)
ECT: Exogenous cueing task
NA: Network analysis
OE: Overeating
EO: Emotional overeating
MeSH: Medical subject headings
EM: Emotional eating (used in some of the included articles)
EDE-Q: Eating disorder examination-questionnaire (EDE-Q)
BES: Binge eating scale
QEWP-5: Eating disorder diagnostic scale and the questionnaire on eating and weight patterns-5

## Declarations

### Protocol

The review protocol can be accessed at HAL (https://hal.science/hal-03643357v1—HAL Id/Registration number: hal-03643357). Protocol reference: Arexis, M., Feron, G., Brindisi, M.-C., Billot, P.-E., & Chambaron, S. (2022). Impacts of emotional regulation and inhibition on Emotional Eating (EE) and Binge Eating Disorder (BED): Protocol for a scoping review. *Hal-03643357*.

## References

[1] van Strien T, van de Laar FA, van Leeuwe JFJ, Lucassen PLBJ, van den Hoogen HJM, Rutten GEHM (2007). The dieting dilemma in patients with newly diagnosed type 2 diabetes: does dietary restraint predict weight gain 4 years after diagnosis?. Health Psychol.

[2] American Psychiatric Association (2013). Diagnostic and statistical manual of mental disorders: DSM-5.

[3] Crockett AC, Myhre SK, Rokke PD (2015). Boredom proneness and emotion regulation predict emotional eating. J Health Psychol.

[4] Dingemans A, Danner U, Parks M (2017). Emotion regulation in binge eating disorder: a review. Nutrients.

[5] Ferrell EL, Watford TS, Braden A (2020). Emotion regulation difficulties and impaired working memory interact to predict boredom emotional eating. Appetite.

[6] Giel KE, Teufel M, Junne F, Zipfel S, Schag K (2017). Food-related impulsivity in obesity and binge eating disorder-a systematic update of the evidence. Nutrients.

[7] Leehr EJ, Krohmer K, Schag K, Dresler T, Zipfel S, Giel KE (2015). Emotion regulation model in binge eating disorder and obesity–a systematic review. Neurosci Biobehav Rev.

[8] Steward T, Menchon JM, Jiménez-Murcia S, Soriano-Mas C, Fernandez-Aranda F (2018). Neural network alterations across eating disorders: a narrative review of fMRI studies. Curr Neuropharmacol.

[9] Waltmann M, Herzog N, Horstmann A, Deserno L (2021). Loss of control over eating: a systematic review of task based research into impulsive and compulsive processes in binge eating. Neurosci Biobehav Rev.

[10] Zhang P, Wu GW, Yu FX, Liu Y, Li MY, Wang Z (2020). Abnormal regional neural activity and reorganized neural network in obesity: evidence from resting-state fMRI. Obes Silver Spring Md.

[11] Greeno CG, Wing RR, Shiffman S (2000). Binge antecedents in obese women with and without binge eating disorder. J Consult Clin Psychol.

[12] Nicholls W, Devonport TJ, Blake M (2016). The association between emotions and eating behaviour in an obese population with binge eating disorder: emotions and binge eating disorder. Obes Rev.

[13] Stein RI, Kenardy J, Wiseman CV, Dounchis JZ, Arnow BA, Wilfley DE (2007). What’s driving the binge in binge eating disorder?: a prospective examination of precursors and consequences. Int J Eat Disord.

[14] Davis C (2013). From passive overeating to « food addiction »: a spectrum of compulsion and severity. ISRN Obes.

[15] Tricco AC, Lillie E, Zarin W, O’Brien KK, Colquhoun H, Levac D (2018). PRISMA extension for scoping reviews (PRISMA-ScR): checklist and explanation. Ann Intern Med.

[16] Arexis M, Feron G, Brindisi MC, Billot PE, Chambaron S. Impacts of emotional regulation and inhibition on Emotional Eating (EE) and Binge Eating Disorder (BED): Protocol for a scoping review. Hal-03643357. 2022.10.1186/s40337-023-00916-737950264

[17] Ouzzani M, Hammady H, Fedorowicz Z, Elmagarmid A (2016). Rayyan—a web and mobile app for systematic reviews. Syst Rev.

[18] Stapleton P, Whitehead M (2014). Dysfunctional eating in an Australian community sample: the role of emotion regulation, impulsivity, and reward and punishment sensitivity. Aust Psychol.

[19] Sultson H, Akkermann K (2019). Investigating phenotypes of emotional eating based on weight categories: a latent profile analysis. Int J Eat Disord.

[20] Kornacka M, Czepczor-Bernat K, Napieralski P, Brytek-Matera A (2021). Rumination, mood, and maladaptive eating behaviors in overweight and healthy populations. Eat Weight Disord EWD.

[21] Deroost N, Cserjési R (2018). Attentional avoidance of emotional information in emotional eating. Psychiatry Res.

[22] Braden A, Musher-Eizenman D, Watford T, Emley E (2018). Eating when depressed, anxious, bored, or happy: are emotional eating types associated with unique psychological and physical health correlates?. Appetite.

[23] Wolz I, Biehl S, Svaldi J (2021). Emotional reactivity, suppression of emotions and response inhibition in emotional eaters: a multi-method pilot study. Appetite.

[24] Wood SMW, Schembre SM, He Q, Engelmann JM, Ames SL, Bechara A (2016). Emotional eating and routine restraint scores are associated with activity in brain regions involved in urge and self-control. Physiol Behav.

[25] Leehr EJ, Schag K, Dresler T, Grosse-Wentrup M, Hautzinger M, Fallgatter AJ (2018). Food specific inhibitory control under negative mood in binge-eating disorder: evidence from a multimethod approach. Int J Eat Disord.

[26] Gianini LM, White MA, Masheb RM (2013). Eating pathology, emotion regulation, and emotional overeating in obese adults with Binge Eating Disorder. Eat Behav.

[27] Walenda A, Kostecka B, Santangelo PS, Kucharska K (2021). Examining emotion regulation in binge-eating disorder. Borderline Personal Disord Emot Dysregulation.

[28] Grant JE, Chamberlain SR (2020). Neurocognitive findings in young adults with binge eating disorder. Int J Psychiatry Clin Pract.

[29] Schag K, Teufel M, Junne F, Preissl H, Hautzinger M, Zipfel S (2013). Impulsivity in binge eating disorder: food cues elicit increased reward responses and disinhibition. PLoS ONE.

[30] Leehr EJ, Schag K, Brückmann C, Plewnia C, Zipfel S, Nieratschker V (2016). A putative association of COMT Val(108/158)met with impulsivity in binge eating disorder. Eur Eat Disord Rev J Eat Disord Assoc.

[31] Aloi M, Rania M, Carbone EA, Caroleo M, Calabrò G, Zaffino P (2021). Metacognition and emotion regulation as treatment targets in binge eating disorder: a network analysis study. J Eat Disord.

[32] Görlach MG, Kohlmann S, Shedden-Mora M, Rief W, Westermann S (2016). Expressive suppression of emotions and overeating in individuals with overweight and obesity. Eur Eat Disord Rev J Eat Disord Assoc.

[33] Racine SE, Horvath SA (2018). Emotion dysregulation across the spectrum of pathological eating: comparisons among women with binge eating, overeating, and loss of control eating. Eat Disord.

[34] Loeber S, Rustemeier M, Paslakis G, Pietrowsky R, Müller A, Herpertz S (2018). Mood and restrained eating moderate food-associated response inhibition in obese individuals with binge eating disorder. Psychiatry Res.

[35] Munsch S, Meyer AH, Quartier V, Wilhelm FH (2012). Binge eating in binge eating disorder: a breakdown of emotion regulatory process?. Psychiatry Res.

[36] Willem C, Gandolphe MC, Doba K, Roussel M, Verkindt H, Pattou F (2020). Eating in case of emotion dys-regulation, depression and anxiety: different pathways to emotional eating in moderate and severe obesity. Clin Obes.

[37] Wang SB, Lydecker JA, Grilo CM (2017). Rumination in patients with binge-eating disorder and obesity: associations with eating-disorder psychopathology and weight-bias internalization. Eur Eat Disord Rev J Eat Disord Assoc.

[38] Mobbs O, Iglesias K, Golay A, Van der Linden M (2011). Cognitive deficits in obese persons with and without binge eating disorder. Investigation using a mental flexibility task. Appetite.

[39] Svaldi J, Naumann E, Trentowska M, Schmitz F (2014). General and food-specific inhibitory deficits in binge eating disorder. Int J Eat Disord.

[40] Kimberlin CL, Winterstein AG (2008). Validity and reliability of measurement instruments used in research. Am J Health-Syst Pharm AJHP Off J Am Soc Health-Syst Pharm.

[41] Frost MH, Reeve BB, Liepa AM, Stauffer JW, Hays RD (2007). Mayo/FDA patient-reported outcomes consensus meeting group; What is sufficient evidence for the reliability and validity of patient-reported outcome measures?. Value Health J Int Soc Pharmacoeconomics Outcomes Res.

[42] Raevuori A, Keski-Rahkonen A, Hoek HW (2014). A review of eating disorders in males. Curr Opin Psychiatry.

[43] Carr MM, Wiedemann AA, Macdonald-Gagnon G, Potenza MN (2021). Impulsivity and compulsivity in binge eating disorder: a systematic review of behavioral studies. Prog Neuropsychopharmacol Biol Psychiatry.

[44] Saruco E, Pleger B (2021). a systematic review of obesity and binge eating associated impairment of the cognitive inhibition system. Front Nutr.

[45] Berchio C, Cambi S, Pappaianni E, Micali N (2022). EEG biomarkers in children and adolescents with feeding and eating disorders: current evidence and future directions. Front Psychiatry.

[46] Turton R, Chami R, Treasure J (2017). Emotional eating, binge eating and animal models of binge-type eating disorders. Curr Obes Rep.

